# Proteomic profiling and correlations with clinical features reveal biomarkers indicative of diabetic retinopathy with diabetic kidney disease

**DOI:** 10.3389/fendo.2022.1001391

**Published:** 2022-10-05

**Authors:** Xiao’e Fan, Manhong Xu, Xin Chen, Qianfeng Ren, Yan Fan, Ranran Wang, Jiaqi Chen, Li Cui, Zhengmin Wang, Xiaoyan Sun, Nannan Guo

**Affiliations:** ^1^ Department of Ophthalmology, Jincheng People’s Hospital, Jincheng, China; ^2^ Department of Vitreoretinal and Ocular Trauma, Tianjin Medical University Eye Hospital, Eye Institute and School of Optometry, Tianjin Key Laboratory of Retinal Functions and Diseases, Tianjin International Joint Research Center of Ophthalmology and Visual Science, Tianjin, China; ^3^ Department of Pathology, Jincheng People’s Hospital, Jincheng, China; ^4^ Department of Laboratory, Jincheng People’s Hospital, Jincheng, China; ^5^ Department of Nephrology, Jincheng People’s Hospital, Jincheng, China

**Keywords:** diabetic kidney disease (DKD), nonproliferative diabetic retinopathy (NPDR), biomarker, proteomics, β-2-Microglobulin (B2M), vimentin (VIM)

## Abstract

Diabetic retinopathy (DR) and diabetic kidney disease (DKD) are complications of diabetes and place serious health and economic burdens on society. However, the identification and characterization of early biomarkers for DKD, especially for nonproliferative DR (NPDR) patients with DKD, are still needed. This study aimed to demonstrate the plasma proteomic profiles of NPDR+DKD and NPDR patients and identify potential biomarkers for early diagnosis of DKD. Fifteen plasma samples from the NPDR group and nine from the NPDR+DKD group were analyzed by LC−MS/MS to identify the differentially expressed proteins between the two groups. Functional enrichment, protein−protein interaction and clinical feature correlation analyses revealed the target protein candidates, which were verified using ELISA and receiver operating characteristic (ROC) analysis. In total, 410 proteins were detected in plasma; 15 were significantly upregulated and 7 were downregulated in the NPDR+DKD group. Bioinformatics analysis suggested that DKD is closely related to cell adhesion and immunity pathways. β-2-Microglobulin (B2M) and vimentin (VIM) were upregulated in NPDR+DKD, enriched as hub proteins and strongly correlated with clinical features. ELISA showed that B2M (p<0.001) and VIM (p<0.0001) were significantly upregulated in NPDR+DKD compared with NPDR. In ROC analysis, B2M and VIM could distinguish DKD from NPDR with area under the curve values of 0.9000 (p < 0.0001) and 0.9950. Our proteomic study revealed alterations in the proteomic profile and identified VIM and B2M as early biomarkers of DKD, laying the foundation for the prevention, diagnosis and treatment of DKD.

## Graphical Abstract

This graphic abstract is original and created by the authors. It has not been published elsewhere.

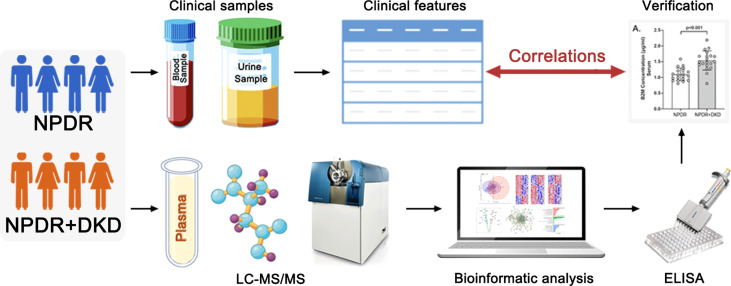

## Introduction

Diabetes mellitus (DM) is a common disease that threatens human health. The World Health Organization survey showed that there were 451 million diabetes patients worldwide in 2017, and this number is expected to increase to 693 million by 2045 (1). The harm mainly lies in the various complications of diabetes, such as diabetic retinopathy (DR), diabetic kidney disease (DKD), and diabetes-induced peripheral neuropathy (DPN), placing serious health and economic burdens on society.

Diabetic retinopathy (DR) is a common severe microvascular complication of diabetes and a common cause of blindness. China has one of the highest prevalence rates of diabetes, and the number of DR patients accounts for 34.6% of the total number of diabetes patients (2); DR causes low vision and even blindness. A five-stage disease severity classification for DR includes three low-risk stages, a fourth stage of severe nonproliferative retinopathy (NPDR), and a fifth stage of proliferative retinopathy (PDR). NPDR is defined as the presence of any of the following: more than 20 intraretinal hemorrhages in each quadrant; definite venous beading in 4 quadrants; prominent intraretinal microvascular abnormalities in 1 or more quadrants; and no signs of proliferative retinopathy. The simplified method of defining severe NPDR is called the “4:2:1 rule”. PDR involves neovascularization and vitreous/preretinal hemorrhage. Eyes with severe NPDR are at high risk for developing PDR (3). Diabetic kidney disease (DKD), also called diabetic nephropathy (DN), is a serious microvascular complication of diabetes. The prevalence and mortality of DKD are increasing annually. DKD is the main cause of end-stage renal disease (ESRD) worldwide, affecting approximately 30% of patients with type 1 DM and approximately 40% of patients with type 2 DM, as reviewed elsewhere (4). DKD is clinically characterized by increased urinary albumin excretion and reduced renal function (5). Clinical diagnosis of DKD is usually based on microalbuminuria (30-300 mg/day) or the urinary albumin/creatinine ratio (>30 mg/g creatinine) (6). However, in some patients with normoalbuminuria, a reduced glomerular filtration rate (GFR) was observed (7), indicating that significant glomerular damage had already occurred before the albuminuria became detectable. A recent classification of DKD stage progression showed that when urinary albumin excretion (UAE) levels are clinically detectable, DKD is already in the third stage (8). Therefore, the identification and characterization of early biomarkers for DKD have become a priority.

DR and DKD are recognized to occur simultaneously in long-term diabetes. The relationship between kidney disease and retinal disease has been discussed in several epidemiological studies (9, 10). However, due to the limitations of current diagnostic methods, DKD cannot be diagnosed soon after onset, especially in patients with DR. Therefore, it is necessary to identify more sensitive and earlier indicators for the identification and diagnosis of DKD in DR patients.

DR and DKD have previously been studied in combination and separately using proteomics (11–13), but to our knowledge, the relationship between DR and DKD has yet not been assessed with plasma proteomics analyses. Here, we detected differentially expressed proteins (DEPs) in the peripheral plasma of NPDR patients with or without DKD by proteomic methods and analyzed the correlation between significant DEPs and clinical DKD diagnostic indicators to identify early biomarkers of DKD and lay the foundation for the prevention, diagnosis and treatment of DKD.

## Materials and methods

### Subjects

Twenty-four subjects were diagnosed with NPDR and recruited for the present study at Jincheng People’s Hospital (Jincheng, Shanxi), of whom nine were diagnosed with DKD. All 24 subjects met the inclusion criteria as follows: (1) The diagnosis and classification of NPDR was based on the standards published by the International Ophthalmological Association (3). (2) Elderly cataract patients with type 2 diabetes mellitus who received phacoemulsification were diagnosed with NPDR. (3) An albumin/creatinine ratio (ACR) ≥ 30 µg/mg in random urine samples occurring at least two out of 3 times within a 3- to 6-month period, according to the 2019 ADA screening and diagnostic criteria (7). All subject who met the above criteria were included in the NPDR+DKD group; those meeting only standard (1) and standard (2) were included in the NPDR group. The exclusion criteria were as follows: (1) proliferative diabetic retinopathy; (2) a history of vitreous hemorrhage, retinal detachment, intraocular surgery (except cataract surgery) or ocular trauma; (3) previous anti-VEGF or laser therapy; (4) complications of uveitis, glaucoma, optic nerve disease or other eye diseases; (5) chronic diseases such as coronary heart disease, hypertension and malignant tumor; (6) type 1 DM, gestational diabetes and special types of diabetes; and (7) an increased ACR due to strenuous exercise, fever, urinary tract infection, nephrotic syndrome or other reasons. The inclusion criteria for DKD were (1) a random urinary albumin-to-creatinine ratio (UACR) ≥30 mg/g or urinary albumin excretion rate (UAER) ≥30 mg/24 h, with repeated assessment of the UACR or UAER within 3 to 6 months, and 2 of the 3 measurements reached or exceeded the critical value; other interfering factors, such as infection, were excluded. (2) The estimated glomerular filtration rate (EGFR) was <60 ml/min/1.73 m^2^ for more than 3 months. (3) Renal biopsy findings were consistent with DKD pathological changes. In accordance with the principles of Declaration of Helsinki and Guidance of Sample Collection of Human Diseases through the Ministry of Public Health of China, informed consent was obtained prior to surgery and sample collection for all participants. Ethical approval for this study was obtained from Jincheng People’s Hospital. Twenty-four subjects were recruited for mass spectrometry analysis at Tianjin Medical University Eye Institute. Another 40 patients were recruited for target protein verification and divided into the NPDR group and the DKD group, each with equal numbers of patients.

### Collection of clinical information

The detailed clinical information for these subjects, including age, sex, body mass index (BMI), mean arterial pressure (MAP), systolic blood pressure (SBP), diastolic blood pressure (DBP) and duration of diabetes, is listed in **Table 1**. Antidiabetic drug therapy have also proved to present several pleiotropic effects, in particular metformin (14). Therefore, the treatments for DM were also listed in **Table 1**. Routine blood and urine tests were performed in the clinical laboratory of Jincheng People’s Hospital. The information was collected and organized by two ophthalmologists and is listed in **Table 2**.

**Table 1:** Characteristics of NPDR and NPDR+DKD patients for plasma collection and LC-MS/MS analysis.

Variables	Total (n=24)	Groups		Statistics	*P*
		NPDR (n=15)	NPDR+DKD (n=9)		
Age(yrs), Mean ± SD	50.50 ± 5.74	50.40 ± 5.73	50.67 ± 6.10	t=-0.11	0.915
Gender, n (%)				-	1.000
Male	18 (75.00)	11 (73.33)	7 (77.78)		
Female	6 (25.00)	4 (26.67)	2 (22.22)		
BMI, Mean ± SD	22.94 ± 1.97	22.88 ± 1.90	23.04 ± 2.19	t=-0.18	0.855
Treatment for DM, n (%)				-	0.615
Oral medication	4 (16.67)	2 (13.33)^a^	2 (22.22)^b^		
Insulin	20 (83.33)	13 (86.67)	7 (77.78)		
MAP, Mean ± SD	127.21 ± 15.86	127.00 ± 15.02	127.56 ± 18.13	t=-0.08	0.936
SBP (mmHg), Mean ± SD	131.79 ± 27.79	128.07 ± 21.15	138.00 ± 36.98	t=-0.84	0.409
DBP (mmHg), Mean ± SD	81.29 ± 13.50	80.27 ± 14.93	83.00 ± 11.34	t=-0.47	0.642
Duration of diabetes (yrs), M (Q_1_,Q_3_)	9.50 (7.00,10.00)	10.00 (7.00,10.00)	9.00 (7.00,10.00)	Z=-0.154	0.878

NPDR, Non-proliferative diabetic retinopathy; NPDR+DKD, Diabetic kidney disease; DM, Diabetes mellitus; BMI, Body Mass Index; MAP, Mean arterial pressure; SBP, Systolic blood pressure; DBP, Diastolic blood pressure; SD, standard deviation; M, Median; Q_1_:1st Quartile; Q_3_:3st Quartile.

a Two patients of NPDR group toke repaglinide.

b Two patients of NPDR group toke repaglinide.

**Table 2:** Clinical features of NPDR and NPDR+DKD patients for LC-MS/MS analysis.

Variables	Total (n=24)	Groups		Statistics	*P*
		NPDR (n=15)	NPDR+DKD (n=9)		
Fasting glucose, M (Q1,Q3)	8.05 (6.85,11.01)	7.81 (6.48,10.84)	9.90 (8.10,11.59)	Z=1.312	*0.189*
HbA1c%, Mean ± SD	9.28 ± 2.06	9.31 ± 2.13	9.22 ± 2.06	t=0.10	*0.919*
TP (g/L), Mean ± SD	66.86 ± 5.41	66.06 ± 6.65	68.19 ± 1.86	t=-1.17	*0.259*
ALB (g/L), Mean ± SD	41.01 ± 5.15	39.86 ± 5.96	42.94 ± 2.72	t=-1.73	*0.099*
UREA, M (Q1,Q3)	5.21 (4.76,6.18)	5.83 (4.98,6.97)	4.84 (4.34,5.34)	Z=-1.789	*0.074*
CRE, M (Q1,Q3)	67.55 (57.85,77.50)	72.90 (63.10,110.10)	61.20 (57.80,76.70)	Z=-0.894	*0.371*
UREA/CRE, M (Q1,Q3)	0.08 (0.06,0.09)	0.07 (0.05,0.09)	0.08 (0.06,0.09)	Z=0.480	*0.631*
UA (umol/L), Mean ± SD	287.59 ± 54.86	298.83 ± 54.40	268.86 ± 53.31	t=1.32	*0.202*
TC, Mean ± SD	4.16 ± 1.00	4.08 ± 1.01	4.28 ± 1.03	t=-0.46	*0.649*
TG, M (Q1,Q3)	1.57 (1.03,2.16)	1.65 (1.03,2.25)	1.27 (1.02,1.58)	Z=-0.865	*0.387*
MA, M (Q1,Q3)	16.50 (7.50,86.00)	9.00 (6.00,65.00)	54.00 (16.00,125.00)	Z=1.402	*0.161*
Ucre, M (Q1,Q3)	5837.00 (4272.50,11108.00)	5567.00 (3978.00,12000.00)	6107.00 (4567.00,10371.00)	Z=-0.119	*0.905*
UACR, M (Q1,Q3)	22.78 (10.40,72.20)	18.00 (8.20,85.00)	35.00 (22.86,66.10)	Z=1.223	*0.221*
WBC, Mean ± SD	6.32 ± 1.58	6.62 ± 1.67	5.82 ± 1.36	t=1.22	*0.235*
RBC, Mean ± SD	4.79 ± 0.94	4.59 ± 1.09	5.14 ± 0.51	t=-1.68	*0.108*
HCT, Mean ± SD	0.42 ± 0.06	0.41 ± 0.07	0.43 ± 0.02	t=-1.06	*0.304*
MCV, Mean ± SD	87.71 ± 4.08	87.40 ± 3.81	88.22 ± 4.68	t=-0.47	*0.643*
MCH, Mean ± SD	30.45 ± 1.34	30.53 ± 1.42	30.32 ± 1.27	t=0.37	*0.717*
PLT, Mean ± SD	225.79 ± 62.22	226.33 ± 59.70	224.89 ± 69.94	t=0.05	*0.958*
MPV, Mean ± SD	10.33 ± 0.98	10.47 ± 1.13	10.09 ± 0.65	t=0.93	*0.364*
PCT, M (Q1,Q3)	0.22 (0.19,0.25)	0.24 (0.20,0.26)	0.20 (0.17,0.22)	Z=-1.465	*0.143*
HDL, Mean ± SD	1.11 ± 0.29	1.03 ± 0.26	1.24 ± 0.30	t=-1.85	*0.077*
LDL, M (Q1,Q3)	2.52 (1.73,3.36)	2.46 (1.69,3.45)	2.70 (1.78,3.10)	Z=0.000	*1.000*
Lpa, M (Q1,Q3)	107.00 (72.50,143.50)	105.00 (76.00,145.00)	117.00 (69.00,143.00)	Z=0.060	*0.952*
APOA, Mean ± SD	1.06 ± 0.23	1.02 ± 0.19	1.13 ± 0.27	t=-1.21	*0.241*
APOB, Mean ± SD	0.86 ± 0.21	0.79 ± 0.18	0.96 ± 0.22	t=-2.02	*0.055*
PT, Mean ± SD	10.98 ± 0.58	11.00 ± 0.64	10.94 ± 0.50	t=0.22	*0.826*
PTA, M(Q1,Q3)	111.50 (103.00,121.50)	106.00 (102.00,116.00)	121.00 (108.00,123.00)	Z=1.403	*0.161*
INR, Mean ± SD	1.02 ± 0.06	1.03 ± 0.04	1.00 ± 0.07	t=1.01	*0.321*
FIB, Mean ± SD	3.99 ± 0.50	4.06 ± 0.43	3.86 ± 0.60	t=0.97	*0.343*
APTT, Mean ± SD	32.13 ± 3.04	32.83 ± 3.40	30.97 ± 1.98	t=1.49	*0.151*
TT, Mean ± SD	15.11 ± 1.47	15.01 ± 1.49	15.28 ± 1.52	t=-0.42	*0.680*

HbA1c, Hemoglobin A1c; TP, Total Protein; ALB, Albumin; CRE, Creatinine; UREA/CRE, urea nitrogen/Creatinine; UA, Urea Acid; TC, Total Cholesterol; TG, Triglycerides; MA, microalbuminuria; Ucre, Urine Creatinine; UACR, Urinary Albumin to Creatinine; WBC, White Blood Cell; RBC, Red Blood Cell; HCT, Hematocrit; MCV, Mean Corpuscular Volume; MCH, Mean Corpuscular Hemoglobin; PLT, Platelet; MPV, Mean Platelet Volume; PCT, Plateletcrit; HDL, High-density Lipoprotein; LDL, Low Density Lipoprotein; Lpa, Lysophosphatidic Acid; APOA, Apolipoprotein A; APOB, Apolipoprotein B; PT, Prothrombin Time; PTA, Prothrombin Activity; INR, International Normalized Ratio; FIB, Fibrinogen; APTT, Activated Partial Thromboplastin Time; TT, Thrombin Time; SD, standard deviation; M, Median; Q_1_,1st Quartile; Q_3_,3st Quartile.

### Proteomic analysis

Blood was collected from the antecubital vein of each patient into an EDTA anticoagulant tube (BD Biosciences, CA, USA) and centrifuged at 1800 × g for 10 min to obtain plasma. Plasma samples were then stored at -80°C until analysis. Blood samples were collected from each patient into individual tubes and used for proteomic analysis. Next, 500 μl of urea lysis buffer containing 8 M urea, 500 mM NH_4_HCO_3_, and 1X protease inhibitor (Roache, Basel, Switzerland) was added to the blood samples for incubation at room temperature (RT) for 5 min, after which the samples were centrifuged at 14,000 rcf for 20 min. Proteins in the supernatant were collected and quantified using a BCA assay (Solarbio, Beijing, China). Then, 100 μg of protein was further lysed and reduced using 10 mM DTT at 37°C for 1 hr and alkylated using 40 nM IAA at 37°C for 1 hr in the dark, and the reaction was terminated using the same concentration of DTT for 1 hr at 37°C. The samples were subsequently washed 3 times using 50 mM NH_4_HCO_3_, eluted with 80 μl of 50 mM NH_4_HCO_3_ in 10 kDa ultrafiltration tubes and digested with 3 μg trypsin at 37°C for 14 hr. The digested samples were washed twice, digestion was terminated with formic acid (FA), and the concentration of the peptides was measured using a Nanodrop 2000 system (Thermo Scientific, MA, USA). Tryptic peptides (4 μg/sample) were then subjected to matrix-assisted laser desorption/ionization (MALDI) mass spectrometry (MS). The liquid chromatography model used in this study was an Ekspert nanoLC 415 (AB SCIEX, MA, USA), and the mass spectrometer was a triple TOF 6600 (AB SCIEX, MA, USA). The digested peptide was loaded into an AB SCIEX trap column (10 × 0.3 mm, C18 packing specification of 5 μm, 120A) with buffer A (0.1% FA, 2% ACN, 97.9% water), and the flow rate was 10 μl/min. The trap column was eluted with different gradients of buffer B (97.9% acetonitrile, 2% water, 0.1% FA). The eluted peptide was passed through the analytical column (150 × 0.3 mm, C18 packing specification of 3 μm, 120A) to form a charged spray and enter mass spectrometry detection. The gradient of buffer B was 0 min-5%, 1 min-6%, 40 min-22%, 52 min-80%, 55 min-80%, 56 min-5%, and 60 min-5%, and the flow rate was 5 μl/min. The mass spectrometry parameters used during data-dependent acquisition (DDA) scanning were as follows: time-of-flight (TOF) MS accumulation time of 0.25 s, mass scanning range of 300-1500 Da, charge selection of +2 - +5 valence ions, mass deviation within 50 ppm, a maximum number of monitored ions in each cycle of 60, isolation of detected ions for 15 s each time, and dynamic fragmentation mode as the fragmentation energy mode. The accumulation time of production was 0.035 s, and the highly sensitive scanning mode was adopted. The mass spectrometry parameters used during data-independent acquisition (DIA) scanning were a TOF MS accumulation time of 0.25 s, a highly sensitive scanning mode in the second stage, 100 variable windows, an accumulation time of 33 ms for each window, and a mass scanning range of 100-1500. The SWATH variable window calculator_ V1.1 program was used to calculate the mass range of each variable window.

### Bioinformatic and statistical analyses

ProteinPilot software (version 5.0.1) was used to search the database for the original data collected in DDA mode. Trypsin was used as the enzyme digestion method. The UniProt human database (including 20368 annotated proteins, released 2020.03) was used as the database. Data with a ProtScore>0.05 were not used. The database search results from ProteinPilot were imported into the SWATH software PeakView (version 2.0) as the database, and the data collected by DIA were quantified. During quantification, the parameters were set, 6 peptide segments were selected for each protein, 6 transitions (ion pairs) were selected for each peptide segment, the peptide confidence was set to 99%, the FDR was set to 1%, modified peptide segments were excluded, the peak extraction window was 10 min, and the quality deviation was within 50 ppm. Two endogenous peptides were selected every 10 minutes to correct the retention time, and the output peak area was used as the quantitative value. At least 2 peptides were required for successful identification.

To process the protein expression data, the median correction was normalized with the original quantitative value using the preprocessCore package in R language, and log2 conversion was performed on the converted data to ensure that it conformed to a normal distribution. The proteins without gene names were removed, the R language stats package t.test function was used to analyze differences in protein expression, and proteins with a p value <0.05 and fold change (FC) >1.5 were screened as the differentially expressed proteins. Gene Ontology (GO), Kyoto Encyclopedia of Genes and Genomes (KEGG) pathway and Reactome pathway analyses were performed using the clusterProfiler package for enrichment analysis. At the same time, the protein−protein interactions of different proteins were analyzed (STRING database, version 11.5, http://string-db.org/).

### Enzyme-linked immunosorbent assays

The target proteins in the plasma from patients were quantified by ELISA kits in an independent cohort consisting of 20 NPDR patients and 20 NPDR patients with DKD. The human VIM ELISA kit (Novusbio, Shanghai, China) and human B2M ELISA kit (Abcam, Shanghai, China) were used to measure the protein concentration in plasma according to the kit instructions, and all samples were tested with two replicates. Briefly, 50 μl of undiluted sample was spotted into a 96-well plate that was precoated with anti-human vimentin antibody, and 50 μl of 20X diluted sample (using the provided dilution buffer) was spotted into a 96-well plate that was precoated with anti-human β-2-microglobulin antibody; both plates were incubated at 37°C for 1 hr. Then, the plates were washed 3 times and incubated with the enzyme-labeled antibody at 37°C; the substrate solution was added to each well, and the plates were incubated for 5 min at 37°C. The reaction was terminated by adding termination solution to each well. The optical density (OD) was measured at 450 nm within 5 min using an Infinite 200 PRO Multimode Microplate Reader (Tecan, Männedorf, Switzerland).

### Receiver operating characteristic analysis

ROC analysis was employed to evaluate the performance of the prognostic model and the clinical parameters, and the R survivalROC package (version 1.0.3; published on 2013-01-13) was used to draw and analyze the ROC curve. Calculation of the areas under the ROC curves was performed to evaluate the accuracy of the model for differentiating NPDR patients with DKD from NPDR patients without DKD.

### Statistical analysis

Continuous data that followed a normal distribution are expressed as the mean ± standard deviation (mean ± SD). Comparison of two datasets was performed with an independent sample t test, and comparisons of multiple datasets were performed using ANOVA. Continuous data with a skewed distribution are presented as the median (lower quartile, upper quartile) [M (Q1, Q3)], comparison of two groups was performed with the rank sum test for two independent samples, and comparisons of multiple groups were performed with the rank sum test (Kruskal−Wallis H test). Categorical data are expressed as the frequency and composition ratio [n (%)] and analyzed with the chi-square test or Fisher’s exact probability method. The correlations between omics and clinical data were analyzed using Spearman rank correlation analysis, and heatmaps were drawn using R4.0.3. Generalized linear models were used to analyze the eye data of the NPDR group and the NPDR+DKD group. Statistical analyses were conducted using SAS 9.4, and differences were considered to be statistically significant at P<0.05.

## Results

### Clinical features of NPDR and NPDR+DKD patients

A previous study showed that the severity of DR is a risk factor for DKD in Chinese patients with T2DM and identified a significant subset of patients with DKD who also had advanced retinopathy, although the severity of the kidney lesions was not always reflected in the severity of DR (15). Therefore, it is essential to predict DKD by observing indicators from patients who come to the hospital for treatment for NPDR. To do so, we collected the plasma of NPDR+DKD and NPDR patients for proteomic detection. The clinical information of the validation cohort is given in **Table 1**. No significant differences were observed in age (p=0.915), sex (p=1.000), BMI (p=0.855), treatment for DM (p=0.615), MAP (p=0.936), SBP (p=0.409), DBP (p=0.642) or duration of diabetes (p=0.878) between the two groups. Because the baseline values of the NPDR and NPDR+DKD groups were comparable, the differentially expressed proteins between these two diseases were more representative and meaningful in subsequent proteomics analysis.

### DEPs between the NPDR+DKD group and NPDR group

**Figure 1:**
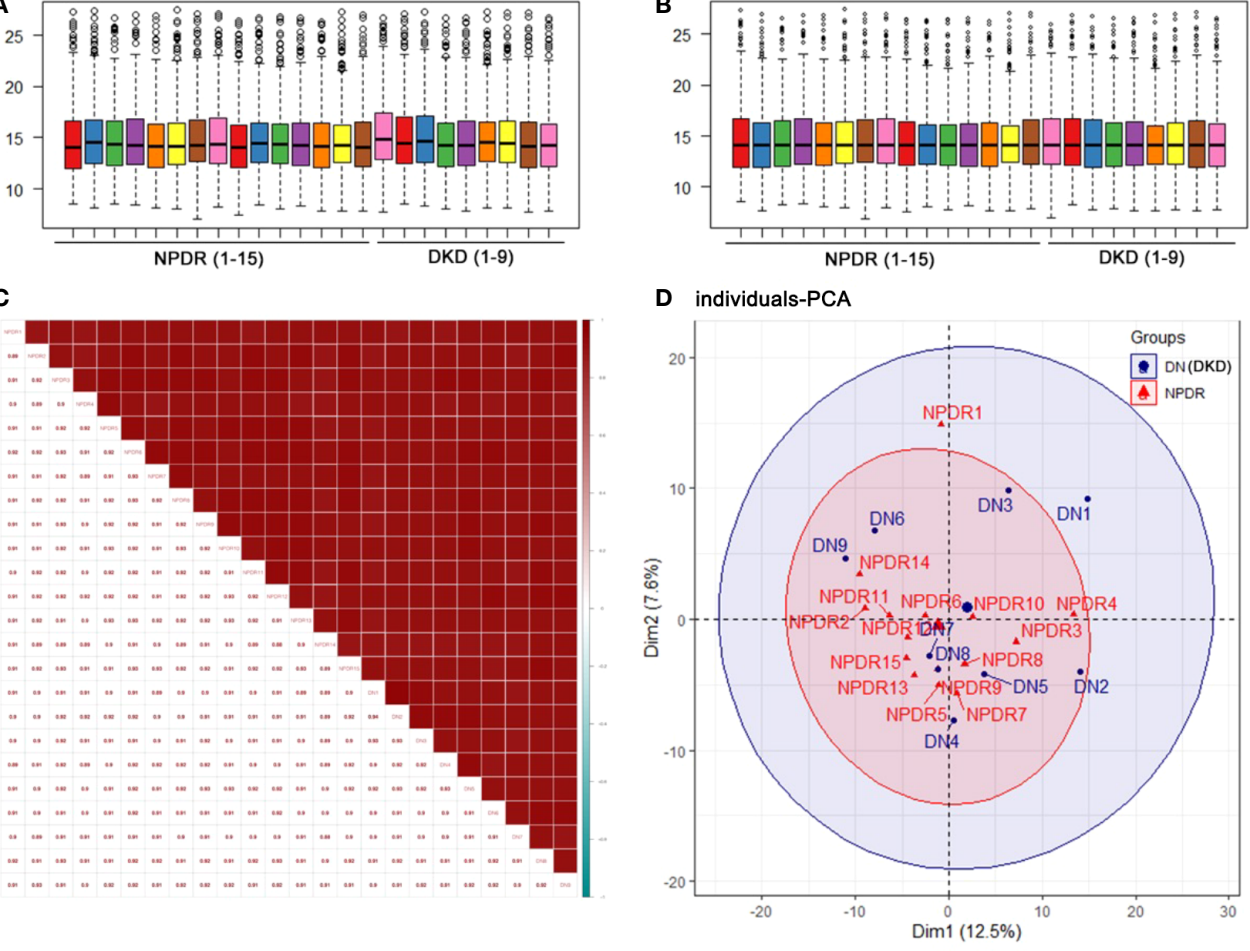
Quality control of the proteomic analysis. **(A)** Median correction before normalization. **(B)** Median correction after normalization. **(C)** Pearson correlation coefficients represent the correlation between samples. A higher correlation and a deeper red color indicate better repeatability of the data. The color bar from red to blue represents correlations from high to low. **(D)** Principal component analysis (PCA) illustrates partial overlap among the NPDR and NPDR+DKD (DKD) groups.

To clarify the differences at the translation level, we performed protein profiling of plasma from a set of 24 samples (including 15 NPDR and 9 NPDR+DKD) by label-free LC−MS/MS. To obtain more reliable results, median correction was normalized (**Figure 1A**) with the original quantitative value using the preprocessCore package in R language, and log2 conversion was performed on the converted data to ensure that it conformed to a normal distribution. The normalized data are shown in **Figure 1B**. The results of Pearson correlation analysis between samples after quality control are shown in **Figure 1C**. The correlation coefficients between samples within and between groups reached 0.88 or above, showing the high repeatability of the data. In addition, principal component analysis (PCA) results showed that the distribution of proteins detected in NPDR+DKD and NPDR samples had some overlap (**Figure 1D**). The distribution of samples in the NPDR group was relatively concentrated, while the NPDR+DKD group had quantitative protein information that the NPDR group did not.

**Figure 2:**
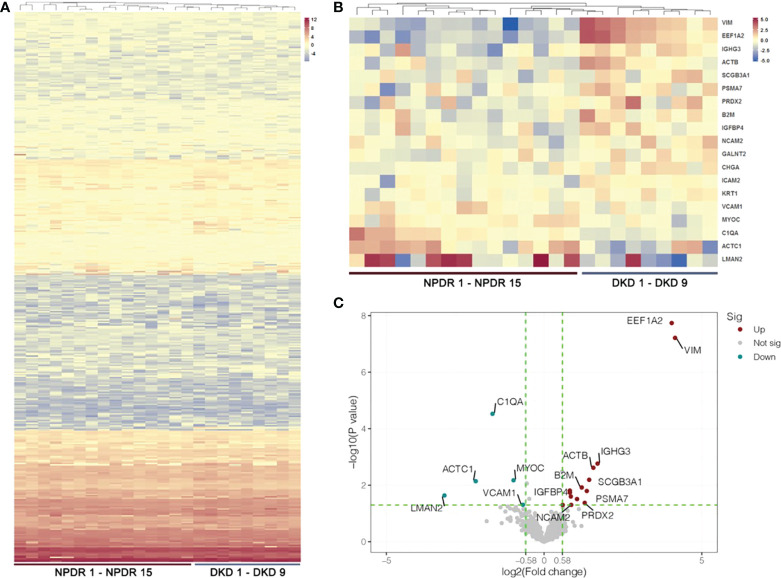
Heatmap and volcano plot of DEPs. **(A)** Hierarchical cluster analysis of all detected proteins in the NPDR and NPDR+DKD (DKD) groups. **(B)** Heatmap showing DEPs between the DKD and NPDR groups. **(C)** The volcano plot represents the DEPs with P<0.05 and FC>1.5 with red and green colors. Red dots show the upregulated proteins, green dots represent the downregulated proteins, and gray dots indicate that the proteins were not significantly up- or downregulated between the DKD and NPDR groups. (n = 15 in NPDR group; n = 9 in DKD group).

With LC−MS/MS analysis, we identified a total of 410 proteins after removing proteins without gene names or proteins with duplicate gene names in the plasma samples of the NPDR and NPDR+DKD groups (**Figure 2A**; **Supplement S1**). Among these proteins, 15 were upregulated, and 7 were downregulated, with a p value<0.05. Over 86% of proteins showed an FC greater than 1.5, including fourteen upregulated proteins and five downregulated proteins in the NPDR+DKD group compared with the NPDR group (**Figure 2B**; **Supplement S2**). The proteins with the highest FC values were VIM (p<0.0001, FC=17.79), eukaryotic translation elongation factor 1 alpha 2 (EEF1A2) (p<0.0001, FC=16.51), IGHG3 (p=0.0017, FC=3.24), ACTB (p=0.0024, FC=2.95), and secretoglobin family 3A member 1 (SCGB3A1) (p=0.006, FC=2.69). The downregulated proteins were lectin mannose-binding 2 (LMAN2) (p=0.023, FC=-8.92), alpha cardiac muscle 1 (ACTC1) (p=0.0072, FC=-4.49), complement C1q subcomponent subunit A (C1QA) (p<0.0001, FC=-3,11), MYOC (p=0.0067, FC=-1.96), and vascular cell adhesion protein 1 (VCAM1) (p=0.0493, FC=-1.59). We highlighted and labeled these proteins in the volcano plot, and data reproducibility is depicted in **Figure 2C**.

### Functional analysis of DEPs

**Figure 3:**
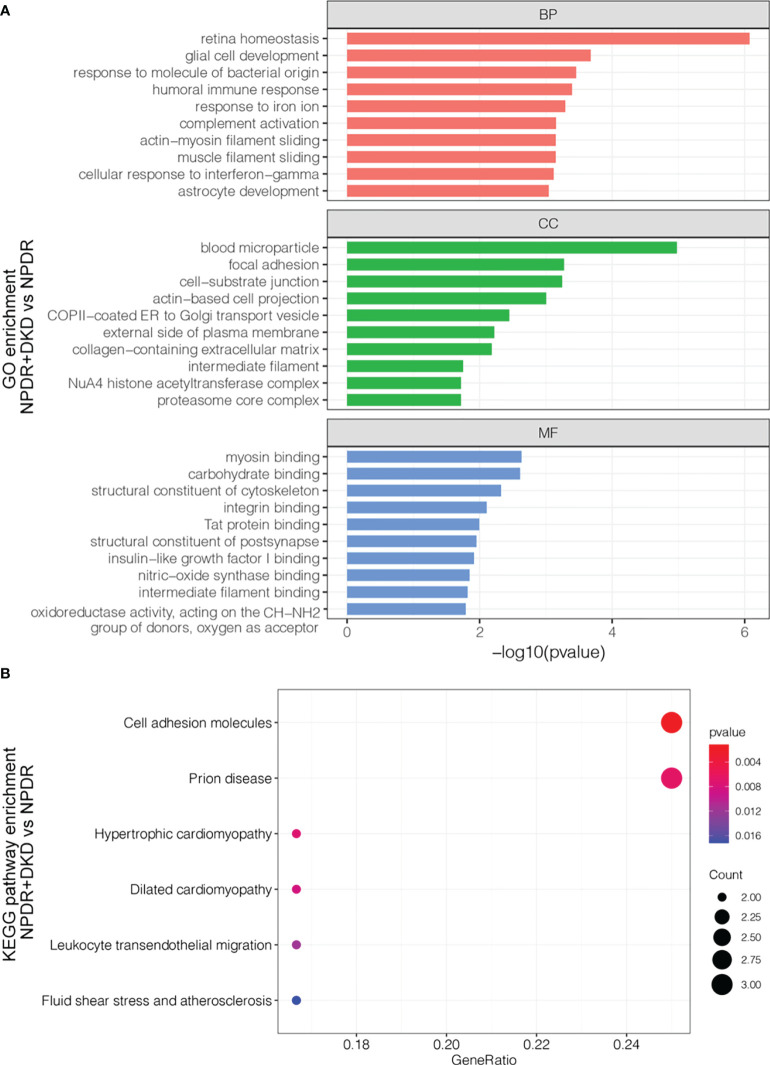
Functional analysis of the DEPs. **(A)** GO analysis of up- and downregulated proteins between NPDR+DKD and NPDR. Only the top 10 terms with the highest -log10(p value) are displayed. BP, biological process; CC, cellular component; MF, molecular function. **(B)** KEGG pathway enrichment. The larger red dot indicates a lower p value and more enriched proteins in that pathway.

Functional analysis of significantly differentially expressed proteins with an FC>1.5 in the plasma samples indicated that they participate in a wide range of biological processes (**Figure 3A**). Biological processes (BPs), such as retina homeostasis, response to molecules of bacterial origin and humoral immune response, were enriched with 4 proteins. Blood microparticles, focal adhesion and cell-substrate junctions were the most highly enriched cellular components (CCs). Differentially expressed proteins between NPDR+DKD and NPDR were mainly involved in the molecular functions (MFs) myosin binding and carbohydrate binding. To further analyze the functionality of DEPs, we also performed KEGG pathway annotation analysis to identify important metabolic and/or signal transduction pathways. Comparison of the NPDR+DKD and NPDR groups showed that there were 6 significant biological pathways. The pathways with the most abundant proteins were cell adhesion molecules and prion disease (**Figure 3B**). ICAM2, NCAM2 and VCAM1 were enriched in the pathway of cell adhesion molecules. C1QA, NCAM2 and PSMA7 were enriched in the prion disease pathway.

**Figure 4:**
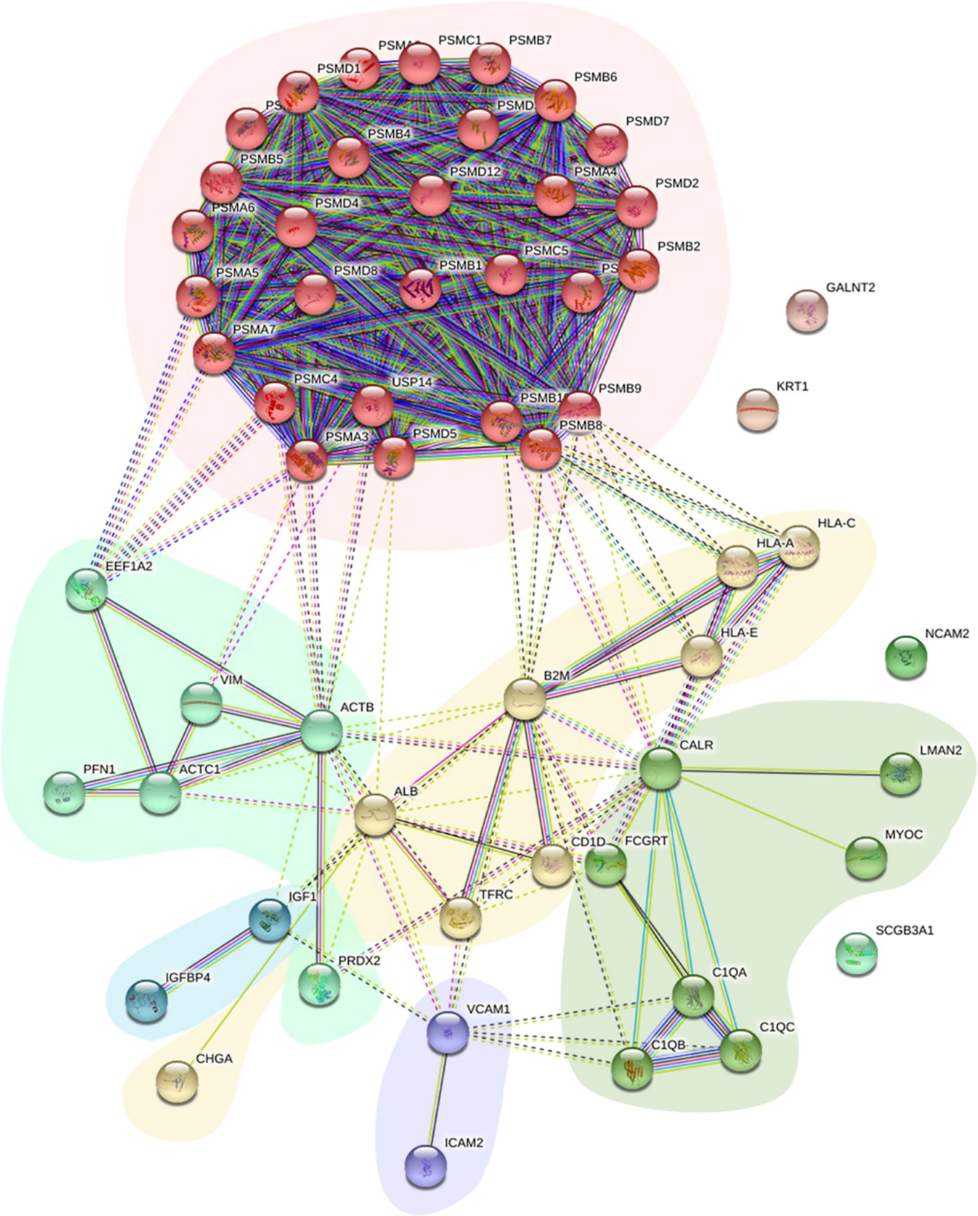
PPI network of DEPs. The PPI network displays the function and signaling connection between the DEPs. Six clusters were generated based on function and labeled with different colors as background.

Protein−protein interactions (PPIs) of the DEPs in the two groups provide clues regarding potential functions. To further explore the relationship between these DEPs from the comparison between NPDR+DKD and NPDR, we used the STRING database and Cytoscape software to evaluate and visualize the PPI analyses. As shown in **Figure 4**, there was close connectivity between each protein pair, especially among centrally located proteins. Six clusters with different background colors are displayed. Proteins in the pink cluster were mainly involved in the proteolytic degradation of most intracellular proteins. Proteins in the yellow cluster were involved in the presentation of peptide antigens to the immune system, with B2M enriched as the hub protein and connected with 12 other proteins. For those in the light green cluster, ACTB and VIM were recognized as hub proteins, and these proteins served as structural proteins and participated in cell migration. In addition, the proteins located in the center of the PPI network were largely involved in retina homeostasis, glial cell development, muscle filament sliding, collagen-containing extracellular matrix, structural constituent of cytoskeleton and intermediate filament binding.

### Clinical features and their correlation with DEPs

In addition to the baseline data presented in **Table 1**, we collected other clinical features of NPDR and NPDR+DKD patients based on clinical tests (**Table 2**). In brief, there were no significant differences between the NPDR+DKD and NPDR groups in terms of fasting glucose (p=0.189), hemoglobin A1c (HbA1c, p=0.919), total protein (TP, p=0.259), albumin (ALB, p=0.099), urea nitrogen (UREA, p=0.074), creatinine (CRE, p=0.371), urea/creatinine (UREA/CRE, p=0.631), uric acid (UA, p=0.202), total cholesterol (TC, p=0.649), triglycerides (TG, p=0.387), microalbuminuria (MA, p=0.161), urine creatinine (Ucre, p=0.905), urinary albumin-to-creatinine ratio (UACR, p=0.221), white blood cell count (WBC, p=0.235), red blood cell count (RBC, p=0.108), hematocrit (HCT, p=0.304), mean corpuscular volume (MCV, p=0.634), mean corpuscular hemoglobin (MCH, p=0.717), platelets (PLT, p=0.958), mean platelet volume (MPV, p=0.364), platelet crit (PCT, p=0.143), high-density lipoprotein (HDL, p=0.077), low density lipoprotein (LDL, p=1.000), lysophosphatidic acid (LPA, p=0.952), apolipoprotein A (APOA, p=0.241), apolipoprotein B (APOB, p=0.055), prothrombin time (PT, p=0.826), prothrombin activity (PTA, p=0.161), international normalized ratio (INR, p=0.321), fibrinogen (FIB, p=0.343), activate partial thromboplastin time (APTT, p=0.151), or thrombin time (TT, p=0.680).

**Figure 5:**
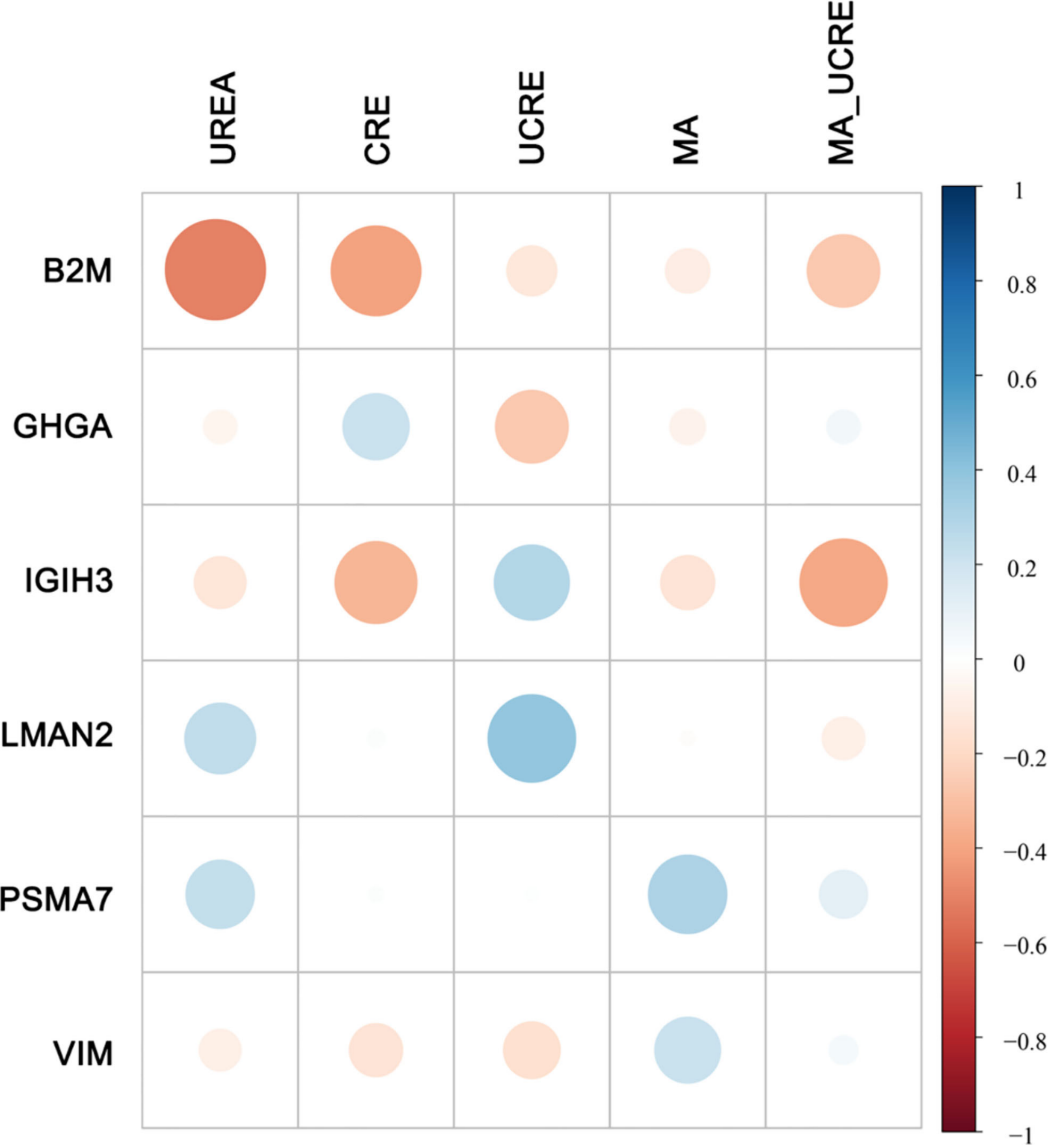
Correlation between DEPs and clinical features. Five clinical features related to DKD diagnosis are listed at the top. The DEPs with the highest number of correlations to the 5 clinical features are displayed on the left. The size of the dot represents the level of relevance. A blue dot represents a positive correlation between the two metrics, and a red dot indicates a negative correlation between the two metrics.

Although there were no significant differences in these clinical features between NPDR+DKD and NPDR, the correlation analysis between the clinical features and DEPs in **Figure 5** shows that some proteins were associated with more than one clinical feature. The indexes, including UREA, CRE, UCRE, MA and MA/UCRE, were closely related to a DKD diagnosis and are listed in **Figure 5**. From all the DEPs, we selected the top 6 with the most correlations to the indexes above. B2M, GHGA, IGIH3 and VIM were correlated with UREA, CRE, UCRE, MA and MA/UCRE; LMAN2 was correlated with UREA, UCRE and MA/UCRE; and PSMA7 was correlated with UCEA, MA and MA/UCRE. Therefore, these proteins may be new indicators for DKD when combined with certain clinical features.

### Target protein selection, validation and clinical significance

**Figure 6:**
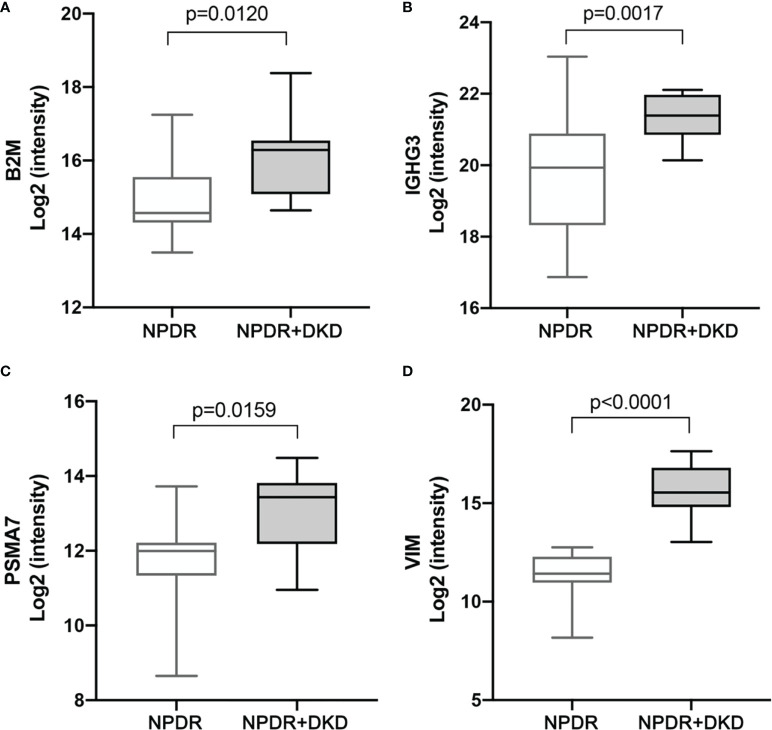
Expression levels of target protein candidates in the plasma of NPDR+DKD vs. NPDR. **(A-D)** Expression levels of B2M, IGHG3, PSMA7 and VIM between the NPDR+DKD and NPDR groups based on proteomics data. (n = 15 in NPDR group; n = 9 in DKD group).

According to the functional, PPI, and correlation analyses, we identified four proteins, B2M, IGHG3, PSMA7 and VIM, and the box diagram of the histological results of these four proteins is shown in **Figure 6**. Based on our proteomics data, the B2M level was significantly increased in NPDR+DKD plasma (P=0.0120), the IGIH3 level was significantly elevated in NPDR+DKD patients (p=0.0017), PSMA7 was significantly upregulated in the NPDR+DKD group (p=0.0159), and the VIM level was also significantly increased in NPDR+DKD samples.

**Figure 7:**
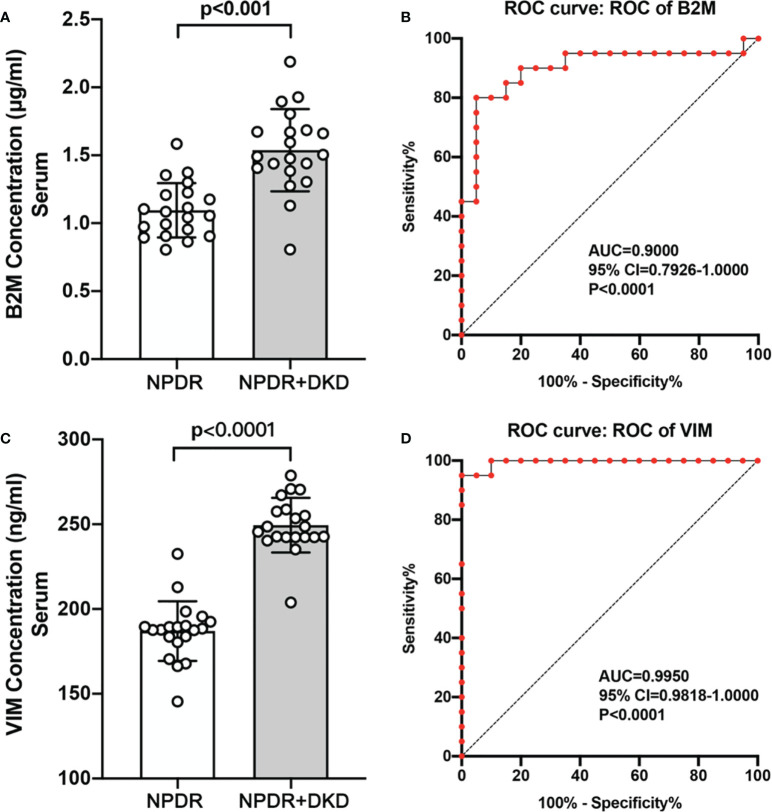
Validation of B2M and VIM by ELISA and ROC analysis. **(A, C)**. The ELISA results showed that the expression of B2M and VIM was significantly upregulated in the NPDR+DKD group compared with the NPDR group. **(B, D)** ROC curves depicting the classification power of B2M and VIM.

To verify the expression of B2M, IGIH3, PSMA7 and VIM in the plasma of NPDR+DKD and NPDR patients, we validated our proteomics results by ELISA with a new set of patients. The plasma samples used for ELISA validation came from 40 new patients (20 in each group). The baseline data are shown in **Table 3**. No significant differences were found in age (p=0.366), sex (p=0.519), BMI (p=0.091), treatment for DM (p=0.342), MAP (p=0.790), SBP (p=0.892), DBP (p=0.413), or duration of diabetes (p=0.690). The ELISA results showed that the expression levels of B2M and VIM in plasma were consistent with the change trend of the proteomic results. B2M expression in the plasma in the NPDR+DKD group (1.538 ± 0.691 μg/ml) was significantly upregulated compared with that in the NPDR group (1.096 ± 0.441 μg/ml) (p<0.001) (**Figure 7A**). The ROC curve depicted the strong discriminatory power of B2M (area under the curve [AUC] =0.9000; p < 0.0001; 95% confidence interval [CI] = 0.7926–1.0000) (**Figure 7B**). The plasma VIM concentration was significantly increased in the NPDR+DKD group (249.4 ± 62.7 ng/ml) compared with the NPDR group (187.0 ± 51.6 ng/ml) (p<0.001) (**Figure 7C**). In addition, the ROC curve depicted the strong discriminatory power of VIM (AUC =0.9950; p<0.0001; 95% confidence interval [CI] = 0.9818–1.0000) for the presence of DKD (**Figure 7D**). The ROC curve illustrated the strong discriminatory power of these two proteins, indicating that VIM and B2M can be considered the distinguishing proteins between NPDR+DKD and NPDR.

**Table 3:** Characteristics of NPDR and NPDR+DKD patients for plasma collection and ELISA.

Variables	Total (n=40)	Groups		Statistics	*P*
		NPDR (n=20)	NPDR+DKD (n=20)		
Age(yrs), Mean ± SD	50.95 ± 9.65	49.55 ± 9.35	52.35 ± 9.99	t=-0.92	0.366
Gender, n (%)				χ^2 =^ 0.417	0.519
Male	24 (60.00)	13 (65.00)	11 (55.00)		
Female	16 (40.00)	7 (35.00)	9 (45.00)		
BMI, Mean ± SD	24.47 ± 2.40	23.82 ± 2.16	25.11 ± 2.51	t=-1.73	0.091
Treatment for DM, n (%)				χ^2 =^ 0.902	0.342
Oral medication	19 (47.50)	8 (40.00)^a^	11 (55.00)^b^		
Insulin	21 (52.50)	12 (60.00)	9 (45.00)		
MAP, Mean ± SD	129.40 ± 6.99	129.70 ± 7.23	129.10 ± 6.91	t=0.27	0.790
SBP (mmHg), Mean ± SD	133.40 ± 15.95	133.05 ± 14.75	133.75 ± 17.46	t=-0.14	0.892
DBP (mmHg), Mean ± SD	83.10 ± 10.24	84.45 ± 12.39	81.75 ± 7.62	t=0.83	0.413
Duration of diabetes (yrs), M (Q_1_,Q_3_)	9.00 (7.00,10.00)	10.00 (7.00,10.00)	9.00 (6.50,10.00)	Z=0.398	0.690

NPDR, Non-proliferative diabetic retinopathy; NPDR+DKD, Diabetic kidney disease; DM, Diabetes mellitus; BMI, Body Mass Index; MAP, Mean arterial pressure; SBP, Systolic blood pressure; DBP, Diastolic blood. pressure; SD: standard deviation; M: Median; Q_1_:1st Quartile; Q_3_:3st Quartile.

a Seven patients of NPDR group toke repaglinide, one patient toke nateglinide.

b Nine patients of NPDR group toke repaglinide, two patients toke nateglinide.

## Discussion

DR and DKD are common microvascular complications of DM. Recently, diabetes treatment had been shifting, in favor of a multifactorial intervention (16). The role of a good quality anamnesis and follow-up is fundamental in order not to misdiagnose in early stage of diseases. It is necessary to find a biomarker of DR+DKD to predict the progression and prognosis of DKD, clarify the pathogenesis and determine the potential therapeutic target of DR+DKD. At present, studies have shown that peripheral plasma proteomics is closely related to the systemic disease associated with DM (12, 13). Since DR and DKD show similar pathological changes and pathogeneses, the DEPs in plasma in this comparison may be closely related to DKD.

In our study, 410 quantifiable proteins were detected in the peripheral plasma; 15 were upregulated, and 7 were downregulated (p<0.05). Through bioinformatics analysis, we found that DEPs were mainly concentrated in blood microparticles, involved in retina homeostasis biological processes, and had myosin binding molecular functions. In addition, we discovered that retinal homeostasis, the humoral immune response, the cellular response to IFN-γ and the astrocyte development pathway may be involved in the inflammatory processes of DR and DKD. Glial cell development, actin-myosin filament sliding, and muscle filament sliding may be involved in the fibrosis process of the retina and kidney in DR and DKD.

The main biological mechanisms of DR and DKD can be linked by the overproduction of superoxide radical oxygen species (ROS), the downstream intracellular signaling pathways and their modulators, which may serve as therapeutic targets for the treatment of diabetic complications. Among these targets, we should mention advanced glycation end products (AGEs) and their receptors, glucose transport molecules, nuclear factor kappa-light-chain-enhancer of activated B cells (NF-κB), protein kinase C (PKC), inflammatory molecules such as adipokines, chemokines, adhesion molecules and proinflammatory cytokines. Furthermore, the profibrotic molecules epidermal growth factor, VEGF, connective tissue growth factors (CTGF) and transforming growth factor beta (TGF-β), which are considered to potentiate the morphological alterations related to diabetic complications, have also been studied (17). Inflammatory molecules, adhesion molecules and proinflammatory cytokines correspond to our GO and KEGG results.

Among the proteins with significant differences in expression between NPDR+DKD and NPDR, those with the highest FC values included VIM, EEF1A2, IGHG3, ACTB and SCGB3A1. VIM is an intermediate filament (IF) protein that participates in epithelial-mesenchymal transition (EMT) (18). EEF1A2 is responsible for the enzymatic delivery of aminoacyl tRNAs to the ribosome, modulating mRNA translation (19). Immunoglobulin heavy constant gamma 3 (IGHG3) is predicted to enable antigen-binding activity and immunoglobulin receptor-binding activity (20). The proteins with the lowest FC values included LMAN2, ACTC1, C1QA, MYOC and VCAM1. LMAN2 encodes a type 1 transmembrane lectin that shuttles between the endoplasmic reticulum, Golgi apparatus and plasma membrane (21) and is associated with the risk of kidney function decline (22). ACTC1 encodes actin, which is involved in various types of cell motility, especially cardiac diseases. C1QA is associated with C1q deficiency and immunodeficiency due to a classical component pathway complement deficiency and has been studied in both retinal and kidney diseases (23, 24).

To further narrow the range of candidate target proteins, we applied PPI network analysis and found 2 proteins, VIM and B2M, located in the network hub. To maximize the clinical significance of target proteins, we then analyzed the correlation between these DEPs and the clinical features. Our results showed that there was no significant difference between NPDR+DKD and NPDR in the results of clinical blood and urine tests, which further verifies that DKD comorbid with DR is hidden and more difficult to detect. However, we were pleasantly surprised to find that 68.2% of the DEPs were related to clinical features, of which 27.3% were related to at least two or more clinical features. More importantly, 6 proteins were found to be related to urine features, and of these, VIM and B2M are hub proteins. Based on this, we believe that VIM and B2M should be considered candidate proteins with high clinical diagnostic significance in DKD patients with DR. Through verification with ELISA, we confirmed that VIM and B2M levels were significantly increased in NPDR+DKD compared with NPDR. The ROC curve depicted the strong discriminatory power of these two proteins for the presence of DKD in DR patients. Therefore, VIM and B2M could be considered potential targets of DKD comorbid with DR based on bioinformatics and validation.

Vimentin, encoded by VIM, a type III intermediate filament protein, is one of the most well-known and extensively studied members of the IF protein family, reflecting their assembly into major cytoskeletal systems in cells of mesenchymal and ectodermal origin. Vimentin is usually considered a mesenchymal marker, and its expression is elevated in Müller glial cells exposed to high glucose conditions in DR (25, 26). Reactive gliosis is characterized by changes in the cell shape due to alterations in intermediate filament production, which include an increase in the expression of vimentin and glial fibrillary acidic protein and decreased expression of glutamine synthetase and glutamate-aspartate transporter in Müller cells in the early stage of DR (27). Vimentin participates in EMT and also exhibits increased levels in the kidney in DKD patients, causing EMT-related renal fibrosis (18, 28). Vimentin regulates the vascular endothelium through the endothelin-NO axis, which has been proven in the renal vascular system. The impairment of angiogenesis and endothelial cell functions in the absence of VIM has also been proven in the retina, by Notch signaling. The TGF-β1-Smad pathway upregulates vimentin expression as mouse alveolar epithelial cells produce the extracellular matrix. There is evidence that vimentin IFs play essential roles in coordinating the signaling pathways that regulate the inflammatory response (29).

β-2-Microglobulin, encoded by B2M, is a small subunit of the major histocompatibility class 1 molecule, which is present on all nucleated cells. Because it is noncovalently associated with the α chain and has no direct attachment to the cell membrane, free B2M circulates in blood after being shed from cell surfaces or by intracellular release. Once released, B2M is almost exclusively eliminated by glomerular filtration and has been used to determine the eGFR. The B2M concentration is fairly constant in healthy individuals, whereas blood levels of B2M increase in disease states such as renal dysfunction and in certain malignancies, autoimmune diseases and infections. Serum B2M has been particularly useful as a clinical marker of chronic kidney disease-related dysfunction (30, 31). B2M is also a tear protein expressed in patients suffering from DR, and its levels have been proven to be elevated by proteomic analysis in NPDR patients compared to healthy volunteers; it is thus considered a possible biomarker of early DR (32). B2M could be considered an early indicator of DR and DKD, but the mechanism still needs to be studied.

In summary, our proteomic study revealed alterations in the proteomic plasma profiles between NPDR+DKD and NPDR patients, in particular validating the roles of VIM and B2M in NPDR patients with DKD. Our results provide new insight into the molecular mechanisms of DKD, especially with regard to EMT and the fibrosis pathway. VIM and B2M have important clinical implications and offer new possibilities for the clinical diagnosis of early-stage DKD, but the mechanism still needs further exploration.

## Data availability statement

The proteomics data has been deposited in ProteomeXchange, as well as in iProX database. The access link of data in proteomexchange is: http://proteomecentral.proteomexchange.org/cgi/GetDataset?ID=PXD036172, ID: PXD036172. The access link of data in iprox is: https://www.iprox.cn/page/project.html?id=IPX0004753000.

## Ethics statement

The studies involving human participants were reviewed and approved by the Ethics Committee of Jincheng People’s Hospital (Approval No: JCPH.No20201026001). The patients/participants provided their written informed consent to participate in this study.

## Author contributions

XF and MX designed the study; MX and XC performed the experiments and wrote the manuscript; QR, YF, RW and JC helped collect the clinical samples; XF, MX and XC analyzed the data; XF provided the funding for the research; ZW, XS and NG helped perform the analysis with constructive discussion. All authors contributed to the article and approved the submitted version.

## Funding

This study was supported by Health Commission of Shanxi Province (No. 2020146), Science and Technology Department of Shanxi Province (No. 202103021223014), and Tianjin Postgraduate Research Innovation Project (2021YJSB271).

## Acknowledgments

The authors thank the surgical team and the nurses of the ophthalmology department of Jincheng People’s Hospital for sample collection. All authors would like to express our gratitude to Mr. Hao Wang, who helped with the bioinformatics analysis and generated the graphs of the proteomics data. All authors would like to thank Mr. Huang, who helped with the statistical analysis. The authors thank Prof.Xiaomin Zhang and Ms.Xuezhi Liang for providing us the authoring environment, space and time for this article.

## Conflict of interest

The authors declare that the research was conducted in the absence of any commercial or financial relationships that could be construed as a potential conflict of interest.

## Publisher’s note

All claims expressed in this article are solely those of the authors and do not necessarily represent those of their affiliated organizations, or those of the publisher, the editors and the reviewers. Any product that may be evaluated in this article, or claim that may be made by its manufacturer, is not guaranteed or endorsed by the publisher.

## Glossary

**Table:**

DR	Diabetic retinopathy
DKD	Diabetic kidney disease
NPDR	Nonproliferative diabetic retinopathy
ROC	Receiver operating characteristic
B2M	β-2-Microglobulin
VIM	Vimentin
DM	Diabetes mellitus
DPN	Diabetes-induced peripheral neuropathy
PDR	Proliferative retinopathy
ESRD	End-stage renal disease
GFR	Glomerular filtration rate
UAE	Urinary albumin excretion
DEPs	Differentially expressed proteins
EGFR	Estimated glomerular filtration rate
UACR	Urinary albumin-to-creatinine ratio
BMI	Body mass index
MAP	Mean arterial pressure
SBP	Systolic blood pressure
DBP	Diastolic blood pressure
GO	Gene Ontology
KEGG	Kyoto Encyclopedia of Genes and Genomes
ELISA	Enzyme-linked immunosorbent assays
ROS	Radical oxygen species
AGEs	Advanced glycation end products
NF-kB	k-light-chain-enhancer of activated B cells
PKC	Protein kinase C
CTGF	Connective tissue growth factors
TGF-b	Transforming growth factor β
EMT	Epithelial-mesenchymal transition
EEF1A2	Eukaryotic translation elongation factor 1 alpha 2
SCGB3A1	Secretoglobin family 3A member 1
LMAN2	Lectin mannose-binding 2
ACTC1	Alpha cardiac muscle 1
C1QA	Complement C1q subcomponent subunit A
VCAM1	Vascular cell adhesion protein 1
PPIs	Protein&minus;protein interactions
IGHG3	Immunoglobulin heavy constant gamma 3
